# Microbial Profile of Tourniquets Used in Phlebotomy at a Rural Tertiary Care Teaching Hospital

**DOI:** 10.7759/cureus.49328

**Published:** 2023-11-24

**Authors:** Arvind Natarajan, Subhashish Das, Nikhil Chaudhary

**Affiliations:** 1 Department of Microbiology, Sri Devaraj Urs Medical College, Sri Devaraj Urs Academy of Higher Education and Research (SDUAHER), Kolar, IND; 2 Department of Pathology, Sri Devaraj Urs Medical College, Sri Devaraj Urs Academy of Higher Education and Research (SDUAHER), Kolar, IND

**Keywords:** hospital acquired infections, microbial flora, multidrug resistance, tourniquet, phlebotomy

## Abstract

Background

Reusable phlebotomy tourniquets may become contaminated through repeated use on the skin surfaces of multiple patients, the hands of healthcare workers, or various surfaces. Noncompliance with the protocol guidelines for managing tourniquets can contribute to the cross-transmission of microorganisms among patients. This study was conducted to determine the microbial flora and antimicrobial sensitivity pattern of reusable phlebotomy tourniquets.

Methodology

Tourniquets were randomly sampled across the different areas of the hospital and were transported to the microbiology laboratory for isolation, identification, and antibiotic susceptibility testing of microorganisms using standard microbiological techniques.

Results

The overall bacterial colonization rate of the 50 tourniquets was 80%. The most prevalent isolate on tourniquets was Coagulase-negative Staphylococcus (9, 22.54%), followed by Micrococcus (6, 15%), Staphylococcus aureus (5, 12.5%), diphtheroid (5, 12.5%), Acinetobacter (4, 10%) Enterococcus (3, 7.5%), Pseudomonas (3, 7.5%), Bacillus (3, 7.5%), and Escherichia coli (2, 5%).

Conclusions

Regular surveillance and disinfection of reusable tourniquets in resource-poor settings are recommended to decrease healthcare infections and the transmission of multidrug-resistant (MDR) strains.

## Introduction

Hospital-acquired infections (HAIs) and antimicrobial resistance are the major global concerns. HAIs cause high costs, increased duration of hospital stay, and mortality among patients [1]. The several potential vectors of transmission of infectious microorganisms include stethoscopes, blood pressure cuffs, pens, mobiles, and tourniquets [2].

Blood collection via peripheral venous access with tourniquets is one of the most common invasive procedures in healthcare settings [3]. It is attributed to the high-pressure level of a tourniquet near the vascular accession site [4].

Reusable phlebotomy tourniquets can become contaminated through repeated use on the skin surfaces of multiple patients, the hands of healthcare workers, or the surfaces on which they are placed [5]. Noncompliance with the protocol guidelines for managing tourniquets can contribute to the cross-transmission of microorganisms among patients [6].

Multidrug-resistant (MDR) bacteria are associated with nosocomial infections and are incumbent menace to public health. The emergence of drug-resistant strains is associated with increased morbidity, mortality, healthcare costs, and antimicrobial use [7].

The present descriptive observational study assessed and determined the microbial contamination and antimicrobial sensitivity pattern of reusable phlebotomy tourniquets from the different healthcare areas of our tertiary care teaching hospital.

## Materials and methods

This descriptive observational study was conducted at R. L. Jalappa Hospital and Research Centre, Kolar, Karnataka, India. Institutional Ethical clearance was obtained with IEC No. DMC/KLR/IEC/569/2022-23.

The tourniquet application time for each patient ranged from 40 to 60 seconds. New tourniquets were used for the study in each ward, with approximately 30 patients included in the analysis. Tourniquets were randomly sampled across the different areas of the hospital (OPDs/wards/ICUs), as shown in Table 1.

**Table 1:** Tourniquets collected from different areas of the hospital. ICU, intensive care unit; OPD, Outpatient Department

Sl. no.	Area	No. of tourniquets
1	Wards	39
2	ICUs	7
3	OPD collection area	2
4	Emergency department	2
	Total	50

The tourniquets were immediately transported to the Central Diagnostic Laboratory Services microbiology section. A direct contact culture approach was employed to simulate the potential hazard of the tourniquet to skin vulnerability as closely as possible. This method was chosen as an alternative to soaking the tourniquet in any enrichment medium, as it might overstate the bacterial load. The tourniquet region likely to be in contact with the patient's skin (1 cm from the buckle) was gently pressed longitudinally across the diameter of a blood agar plate using an aseptic method [8]. The culture plates were incubated aerobically at 37 °C for one to two days. Isolation, identification, and antibiotic sensitivity testing of the organisms were done by standard microbiological techniques and Clinical Laboratory Standard Institute (CLSI) guidelines [9].

Identification of the isolates

Identical colonies of the growth were chosen for microscopic and biochemical tests. Gram stain was employed for the microscopic examination. The biochemical tests carried out included catalase, coagulase, pyrazinamidase, bile aesculin, oxidase, motility, triple sugar iron, mannitol fermentation, urease, citrate, and indole tests, as mentioned in Table 2.

**Table 2:** Identification of bacteria. TSI, triple sugar iron

Sl. no.	Bacteria	Gram stain	Colony morphology	Biochemical test
1	Coagulase-negative Staphylococcus (CoNS)	Gram-positive cocci in clusters	Non-hemolytic white colonies	Catalase test positive, coagulase test negative
2	Micrococcus	Gram-positive cocci in tetrads	Cream- to yellow-colored colonies	Resistance to Mupirocin, sensitive to Bacitracin
3	Diphtheroid	Gram-positive bacilli with palisade arrangement	Non-hemolytic colonies	Catalase test positive, pyrazinamidase test positive
4	Staphylococcus aureus	Gram-positive cocci in clusters	Hemolytic golden yellow colonies	Catalase test positive, coagulase test positive
5	Bacillus	Gram-positive bacilli	Hemolytic white colonies	Catalase test positive, motility test positive, penicillin resistance
6	Enterococcus	Gram-positive cocci in pairs and short chains	Non-hemolytic gray colonies	Catalase test negative, bile aesculin positive
7	Acinetobacter spp.	Gram-negative coccobacilli	Non-lactose fermenters	Catalase test positive, oxidase negative, TSI: inert, citrate positive, urease negative, indole negative, mannitol negative, motility test negative
8	Pseudomonas spp.	Gram-negative bacilli	Non-lactose fermenters	Catalase test positive, oxidase negative, TSI: inert, citrate positive, urease negative, indole negative, mannitol negative, motility test positive
9	Escherichia coli	Gram-negative bacilli	Lactose fermenters	Catalase positive, indole positive, TSI: acidic slant/acidic butt, citrate negative, urease negative, mannitol positive, motility test positive

Antimicrobial sensitivity testing (AST)

AST was performed on Mueller Hinton agar for all bacterial isolates using the modified Kirby Bauer disc diffusion technique according to the latest CLSI guidelines [10].

Statistical methods

Data were entered into a Microsoft Excel sheet and analyzed using IBM SPSS Statistics for Windows, version 22 (IBM Corp., Armonk, NY) software. The values are expressed in frequency and percentages.

## Results

Of the 50 tourniquets collected, 29 (58%) were from wards, 7 (14%) from ICUs, 2 (4%) from the OPD collection area, and 2 (4%) from the emergency department.

Out of 50 tourniquet samples, 40 (80%) yielded positive growth, of which 23 (57.5%) showed nonpathogenic opportunistic colonizers and 17 (42.5%) yielded potentially pathogenic bacteria.

Among 40 positive cultures, 31 (77.5%) were Gram-positive bacteria of which coagulase-negative Staphylococcus (CoNS, 9, 22.5%) was predominant, followed by Micrococcus (6, 15%), Staphylococcus aureus (5, 12.5%), diphtheroid (5, 12.5%), Enterococcus (3, 7.5%), and Bacillus (3, 7.5%). Among 9 (22.5%) Gram-negative bacteria, Acinetobacter was predominant (4, 10%) followed by Pseudomonas (3, 7.5%) and Escherichia coli (2, 5%), as shown in Table 3.

**Table 3:** Microbial colonization of tourniquets. ICUs, intensive care units; OPD, outpatient department, CoNS, coagulase-negative staphylococcus; spp., species

Sl. no.	Microorganisms	Wards (*n* = 31)	ICUs (*n *= 7)	OPD collection (*n *= 2)	Emergency department (*n *= 2)	Total
1	CoNS	6	1	1	1	9 (22.5%)
2	Micrococcus	4	1	1	-	6 (15%)
3	Diphtheroid	4	-	-	1	5 (12.5%)
4	Staphylococcus aureus	3	2	-	-	5 (12.5%)
5	Bacillus	3	-	-	-	3 (7.5%)
6	Enterococcus	2	1	-	-	3 (7.5%)
7	Acinetobacter spp.	2	2	-	-	4 (10%)
8	Pseudomonas spp.	2	1	-	-	3 (7.5%)
9	Escherichia coli	2	-	-	-	2 (5%)
Total microbes isolated from tourniquets from different areas		29	7	2	2	40

In our study, 4 (10%) bacteria showed multidrug resistance, i.e., resistance to more than three classes of antibiotics. Methicillin-resistant Staphylococcus aureus (MRSA) was detected in 2 (5%) strains of S. aureus, and 2 (5%) of Acinetobacter species were MDR, and all these four MDR strains were isolated from ICU tourniquets.

## Discussion

Our study revealed an overall bacterial colonization rate of 80% (40) from sampled tourniquets. Our findings are consistent with a study conducted by Ogba et al., which revealed a bacterial colonization rate of 85% [11].

The study by Kalyani et al. showed a bacterial colonization rate of 100% [12]. In contrast, the study conducted by Zara et al. reported a bacterial colonization rate of 51.0%, which was lower than our study [13]. Several studies have reported that microbiological contamination in tourniquets varied between 9% and 100% [11-13].

Our study also showed higher contamination of the tourniquets by Gram-positive (31, 77.5%) compared to Gram-negative (9, 22.5%) types. In our study, Gram-positive bacteria preponderantly comprised CoNS (9, 22.5%) followed by Micrococcus (6, 15%), S. aureus (5, 12.5%), diphtheroid (5, 12.5%), Enterococcus (3, 7.5%), and Bacillus (3, 7.5%). This may be due to the improved endurance of Gram-positive bacteria in opposition to Gram-negative bacteria [14].

CoNS species (9, 22.5%) were isolated predominantly in our study, which could be attributed to patients' indigenous flora and their higher capability to survive for prolonged periods in tourniquets. Our findings, indicating CoNS as the predominant isolates from the tourniquets, align with those of other investigators. CoNS were highly represented in their studies as well [5,15,16].

In our study, Gram-negative bacilli predominantly comprised Acinetobacter, followed by Pseudomonas and Escherichia coli. A study by Donna et al. also reported Acinetobacter as the predominant bacteria among the Gram-negative type [17]. In contrast to our findings, other studies have reported Enterobacteriaceae as predominant among Gram-negative bacteria [5].

In our study, out of 40 (80%) positive bacterial growth, 23 (57.5%) of the bacterial isolates appeared to be of low pathogenicity and almost consistent with normal skin flora and 7 (17.5%) were environmental isolates.

In our study, 4 (10%) bacteria showed multidrug resistance. MRSA was detected in two (5%) strains of S. aureus. Several studies have also reported tourniquets contaminated with MRSA [18,19]. MRSA infections pose a serious health concern due to the limited therapeutic options available and the challenges associated with eradicating them from hospital settings [20]. All three Enterococci isolated from our study were sensitive to vancomycin.

In our study of Gram-negative bacilli colonization, 2 (5%) of Acinetobacter species also exhibited multidrug resistance. Acinetobacter can survive for longer duration on environmental surfaces if they are improperly cleaned [21].

Cross-infection with Acinetobacter baumannii in hospital settings has led to nosocomial outbreaks associated with high mortality [22]. Pinto et al. also reported colonization by MDR Gram-negative organisms with transmissible β-lactamase enzymes [23].

Different studies demonstrate that extremely portable medical gadgets are related to higher contamination rates, frequently coupled with bacterial isolates that are MDR strains. The tourniquets can act as a potential reservoir, and there may be a risk of transmitting potential pathogens. As their place of use is adjacent to the blood drawing site, some microorganisms existing there can be accountable for any bloodstream infections [24].

Infection prevention and control practices are emphasized in all hospital areas, including hand hygiene and decontamination between procedures. The lack of disinfection between each patient contact may be attributed to factors such as work pressure, a lethargic attitude, negligence, and the challenges associated with disinfecting elastic fabric tourniquets [25].

To prevent the cross-transmission of microorganisms, tourniquets should be manufactured using materials with a lower risk of bacterial contamination. The present guidelines advocate the use of single-patient tourniquets, and in the case of low-resource settings, it is mandatory to disinfect tourniquets between each patient contact [26,27,28].

The limitations of this study include the relatively small sample size, and the colony-forming units of the isolates were not determined.

## Conclusions

Reusable phlebotomy tourniquets are used repeatedly on multiple patients and can serve as potential sources of cross-infection between patients due to the presence of pathogenic bacteria. To prevent cross-infection among patients, it is advisable to use a single-use disposable tourniquet.

The tourniquet used in our study may act as a source for the contagion of bacteria, posing a potential threat to the safety and quality of patient care services.

Regular surveillance and disinfection of reusable tourniquets in resource-poor settings are recommended in Infection Control programs to decrease healthcare infections and transmission of MDR strains. Hand hygiene is an essential, cost-effective measure that, if practiced regularly, can reduce the transmission of microorganisms.
